# Identification of an IRF10 gene in common carp (*Cyprinus carpio* L.) and analysis of its function in the antiviral and antibacterial immune response

**DOI:** 10.1186/s12917-020-02674-z

**Published:** 2020-11-19

**Authors:** Yaoyao Zhu, Shijuan Shan, Huaping Zhao, Rongrong Liu, Hui Wang, Xinping Chen, Guiwen Yang, Hua Li

**Affiliations:** 1 Shandong Provincial Key Laboratory of Animal Resistance Biology, College of Life Sciences, Shandong Normal University, No. 88 East Wenhua Road, Jinan, 250014 China; 2 College of Fisheries and Life Science, Hainan Tropical Ocean University, No. 1 Yucai Road, Sanya, 572022 China

**Keywords:** Common carp (*Cyprinus carpio* L.), Interferon regulatory factor 10 (IRF10), poly I:C, *Aeromonas hydrophila*, IFN response

## Abstract

**Background:**

Interferon (IFN) regulatory factors (IRFs), as transcriptional regulatory factors, play important roles in regulating the expression of type I IFN and IFN- stimulated genes (ISGs) in innate immune responses. In addition, they participate in cell growth and development and regulate oncogenesis.

**Results:**

In the present study, the cDNA sequence of IRF10 in common carp (*Cyprinus carpio* L.) was characterized (abbreviation, *Cc*IRF10). The predicted protein sequence of *Cc*IRF10 shared 52.7–89.2% identity with other teleost IRF10s and contained a DNA-binding domain (DBD), a nuclear localization signal (NLS) and an IRF-associated domain (IAD). Phylogenetic analysis showed that *Cc*IRF10 had the closest relationship with IRF10 of *Ctenopharyngodon idella*. *Cc*IRF10 transcripts were detectable in all examined tissues, with the highest expression in the gonad and the lowest expression in the head kidney. *Cc*IRF10 expression was upregulated in the spleen, head kidney, foregut and hindgut upon polyinosinic:polycytidylic acid (poly I:C) and *Aeromonas hydrophila* stimulation and induced by poly I:C, lipopolysaccharide (LPS) and peptidoglycan (PGN) in peripheral blood leucocytes (PBLs) and head kidney leukocytes (HKLs) of *C. carpio*. In addition, overexpression of *Cc*IRF10 was able to decrease the expression of the IFN and IFN-stimulated genes PKR and ISG15.

**Conclusions:**

These results indicate that *Cc*IRF10 participates in antiviral and antibacterial immunity and negatively regulates the IFN response, which provides new insights into the IFN system of *C. carpio*.

**Supplementary Information:**

The online version contains supplementary material available at 10.1186/s12917-020-02674-z.

## Background

Interferon (IFN) regulatory factors (IRFs), as transcriptional regulatory factors, play multiple important roles in host immune responses and other physiological processes; for example, they regulate the expression of type I IFN and IFN-stimulated genes (ISGs) [1], the activation and differentiation of distinct immune cell populations, cell growth, differentiation and apoptosis [2, 3]. The N-terminal regions of IRFs share a highly conserved DNA-binding domain (DBD), which contains five or six tryptophan repeats [4]. The C-terminal regions of IRFs generally possess an IRF-associated domain (IAD), which mediates the interactions between IRFs and other proteins to form transcriptional complexes [5].

To date, 11 kinds of IRFs have been identified in vertebrates [6], which can be divided into four subfamilies, IRF1 (IRF1, 2, and 11), IRF3 (IRF3 and 7), IRF4 (IRF4, 8, 9, and 10) and IRF5 (IRF5 and 6), according to their differences in the C-terminal region [7]. IRF10 is present only in non-mammals and was first identified in the chicken (*Gallus gallus*). Phylogenetic analysis has shown that *Gg*IRF10 clusters into the IRF4 subfamily and plays a crucial role in the later stages of the immune response to invading pathogens in *G. gallus* [8]. Functional characterization of fish IRF10 has also begun recently, and this gene has been identified in teleosts including zebrafish (*Danio rerio*) [9], orange spotted grouper (*Epinephelus coioides*) [10, 11], Japanese flounder (*Paralichthys olivaceus*) [12], Atlantic cod (*Gadus morhua*) [13, 14], grass carp (*Ctenopharyngodon idella*) [15], rainbow trout (*Oncorhynchus mykiss*) [15, 16], Asian swamp eel (*Monopterus albus*) [15, 17], mandarin fish (*Siniperca chuatsi*) [18], Senegalese sole (*Solea senegalensis*) [19] and blunt snout bream (*Megalobrama amblycephala*) [20].

The teleost IRF10 genes are constitutively expressed in a variety of tissues and can be upregulated by the viral mimic polyinosinic:polycytidylic acid (poly I:C) [10–15, 17, 18, 21], lipopolysaccharide (LPS) [12, 17], viruses [12, 18, 19] or bacteria [10, 12, 13, 17, 20], suggesting that IRF10 plays roles in immune responses to both viral and bacterial infections. Furthermore, in zebrafish and orange spotted grouper, IRF10 inhibits IFNφ1 and IFNφ3 promoter activation and negatively regulates fish antiviral gene expression to prevent an excessive immune response, which is a unique regulatory mechanism of IFN responses in teleosts [10, 11].

As a pivotal aquaculture fish species widely cultured in both Asia and Europe, common carp (*Cyprinus carpio* L.) is also a good model for exploring the immune system of teleosts [22–24]. Several IRFs have been identified in common carp, including IRF1, IRF3, IRF5, IRF7 and IRF9 [25–28]. In this study, we identified the full-length cDNA sequence of IRF10 in *C. carpio* (named *Cc*IRF10) and investigated its function. We examined the tissue distribution of *Cc*IRF10 in healthy common carp and then evaluated the expression level of *Cc*IRF10 upon viral or bacterial stimulation both in vivo and in vitro to determine its function in the immune response against pathogens. Furthermore, the regulatory role of *Cc*IRF10 in the IFN signalling pathway was also determined in this study. The results will contribute to understanding of the innate immune system of fish.

## Methods

### Fish feeding and sampling

*C. carpio* specimens were purchased from a local fish farm (Jinan, Shandong, China), selected for size (approximately 200 g per fish), maintained in recirculating tap water at 20 °C and fed daily for more than 1 week before challenge and sampling. After treatment, the fish were euthanized by immersion in a solution of tricaine methane sulfonate (MS222, Sigma-Aldrich) at a concentration of 100 mg/l of water.

### Cloning and analysis of *Cc*IRF10 cDNA

The full-length cDNA sequence of *Cc*IRF10 was obtained using RT-PCR and the rapid amplification of cDNA ends (RACE) method. First, the primers IRF10-F/IRF10-R were used to amplify a partial sequence of *Cc*IRF10. The primers were designed based on the known fish IRF10 cDNA sequences. Then, 3′ and 5′ Full RACE Core Sets (TaKaRa) were used to obtain the full-length cDNA sequence. The PCR products were ligated into the pMD18-T vector (TaKaRa) and transformed into competent *E. coli* DH-5α for sequencing (Invitrogen). The domains of the protein sequence were analysed using the Simple Modular Architecture Research Tool (SMART, http://smart.embl-heidelberg.de). Multiple alignment and phylogenetic analysis were performed using MEGA 5.0. The primers used in this study are listed in Table 1.

**Table 1:** Primers used in the present study

Name of primer	Sequence(5′-3′)	GenBank accession No.
*Cc*IRF10-F	TAGCGCAGATAGACAGCG	MT646905
*Cc*IRF10-R	CACACCTTTCTCCAGGTG	
*Cc*IRF10 ORF-F	CCGGAATTCATGGAAGACAGGTCGAGGCA	
*Cc*IRF10 ORF-R	TCCCCGCGGTTCACTGGTTTTCCTGTGTGG	
*Cc*IRF10 RT-F	GCTGTTGGATGGAGTGTGAATGG	
*Cc*IRF10 RT-R	CCAGGTTCCCGTGATAGAACAAAC	
*Cc*S11-F	CCGTGGGTGACATCGTTACA	
*Cc*S11-R	TCAGGACATTGAACCTCACTGTCT	
EPC-IFN-F	TCAATCTCATGGATGCCTCAGAGC	FN178457
EPC-IFN-R	TGGTATTGGGCCACGCATTCTT	
EPC-TNFα-F	ACAGGTGATGGTGTCGAGGAGGA	JN412133
EPC-TNFα-R	TCTGAGACTTGTTGAGCGTGAAG	
EPC-ISG15-F	GTGAGCGGTGAAGCCACAGTTG	KM099174
EPC-ISG15-R	GCGAACCGTTATCGGCAGACAG	
EPC-PKR-F	AGGCTTGATCCACAGAGACCTGAA	KM099176
EPC-PKR-R	CGTTCCAGAAGTTGCACGTCATTG	
EPC-EF1α-F	AAGAGCGTTGAGAAGAAAG	AY643400
EPC-EF1α-R	GAGTGCCCAGGTTTAGAG	

### Experimental challenge of *C. carpio* with poly I:C and *Aeromonas hydrophila*

Poly I:C and *A. hydrophila* challenge experiments were performed in *C. carpio* as previously described [28–30]. In the poly I:C challenge experiments, 50 fish were intraperitoneally injected with 500 μl of poly I:C solution (2.6 mg/ml in PBS, Sigma). In the *A. hydrophila* challenge experiment, the bacteria were inactivated in 0.5% formalin at 37 °C for 36 h and then resuspended in PBS. Then, 50 fish were intraperitoneally injected with 500 μl of *A. hydrophila* (at a dose of 2.0 × 10^8^ cells). At 0, 3, 6, 12, 24, 48 and 72 h post injection (hpi), the spleen, head kidney, foregut and hindgut were collected from three fish at every time points (*n* = 3). Total RNA was extracted using TRIzol reagent (TIANGEN), and cDNA was synthesized using a FastQuant RT Kit (TIANGEN).

### In vitro stimulation of PBLs and HKLs

Peripheral blood leucocytes (PBLs) and head kidney leukocytes (HKLs) were isolated from *C. carpio* according to a previous report [28, 31]. In brief, *C. carpio* peripheral blood and head kidney cell suspensions were loaded onto freshly prepared 34%/51% Percoll (Sigma) density gradients and separated via centrifugation at 650×*g* for 30 min. The cells were resuspended in cold Leibovitz’s L-15 medium with 10% foetal bovine serum (FBS), 100 UI/ml penicillin and 100 mg/ml streptomycin. PBLs or HKLs (1 × 10^6^) were maintained in a 24-well cell culture plate with 500 μl in each well and treated with 5 μl poly I:C (500 μg/ml), LPS (1 mg/ml) or peptidoglycan (PGN, 1 mg/ml). At 0, 3, 6, 12 and 24 h post stimulation, triplicate cell samples were harvested, and total RNA was extracted (*n* = 3).

### Construction and transfection of *Cc*IRF10 overexpression vectors

The ORF of *Cc*IRF10 was amplified using Phusion High-Fidelity DNA polymerase (PrimeSTAR) with specific primers. Purified fragments were digested with the SacII and EcoRI restriction enzymes, ligated into the pcDNA3.1-EGFP vector, and transformed into *E. coli* Top10 cells. The overexpression vector pcDNA3.1-EGFP-*Cc*IRF10 (abbreviation, pcIRF10) was verified by sequencing. The plasmids were extracted using an endotoxin-free plasmid isolation kit (TIANGEN) following the manufacturer’s instructions.

Epithelioma papulosum cyprini (EPC) cells were seeded in 24-well plates with 500 μl in each well at a concentration of 4 × 10^5^ cells/ml and maintained at 25 °C in M199 medium (HyClone) supplemented with 10% FBS, 100 U/ml penicillin and 100 μg/ml streptomycin (Gibco) for 1 day so that they reached approximately 80% confluency. Each well of cells was transfected with 1 μg of plasmids using 2 μl of X-tremeGENE HP DNA Transfection Reagent (Roche). EPC cells transfected with empty plasmids served as controls, and the gene expression of IFN, PKR, ISG15 and TNFα was detected after overexpression of *Cc*IRF10. The primers used in this study are listed in Table 1.

### Real-time PCR analysis

Real-time PCR was performed as previously described in a Rotor-Gene Q PCR instrument (Qiagen) with TransStart Tip Green qPCR SuperMix (TransGen) [28]. All samples were analysed in triplicate, and the expression values of all genes were calculated relative to those of the 40S ribosomal protein S11 or the β-actin gene using the 2^(−∆∆CT)^ method. The primers used are listed in Table 1.

### Statistical analysis

Comparisons between the experimental group and the control group were performed using one-way analysis of variance (ANOVA) in GraphPad Prism 5, and a value of *P* < 0.05 was considered to indicate significance.

## Results

### cDNA cloning and molecular characterization of *Cc*IRF10

The full-length cDNA of *Cc*IRF10 was found to consist of 1274 bp. The *Cc*IRF10 cDNA (GenBank accession no. MT646905) contains a 78 bp 5′-untranslated region (UTR), a 26 bp 3′-UTR containing mRNA instability motifs (^1244^AATAA^1249^), and an ORF of 1170 bp that translates into a 390-amino acid putative peptide with a predicted molecular mass of 44.4 kDa. The theoretical isoelectric point is 8.314. The predicted protein sequence contains a DBD (M1-R119) that possesses five tryptophans (Trp13, Trp28, Trp40, Trp60 and Trp79), an IAD (P186-L367) and a nuclear localization signal (NLS) in the DBD (Fig. S1).

**Fig. 1:**
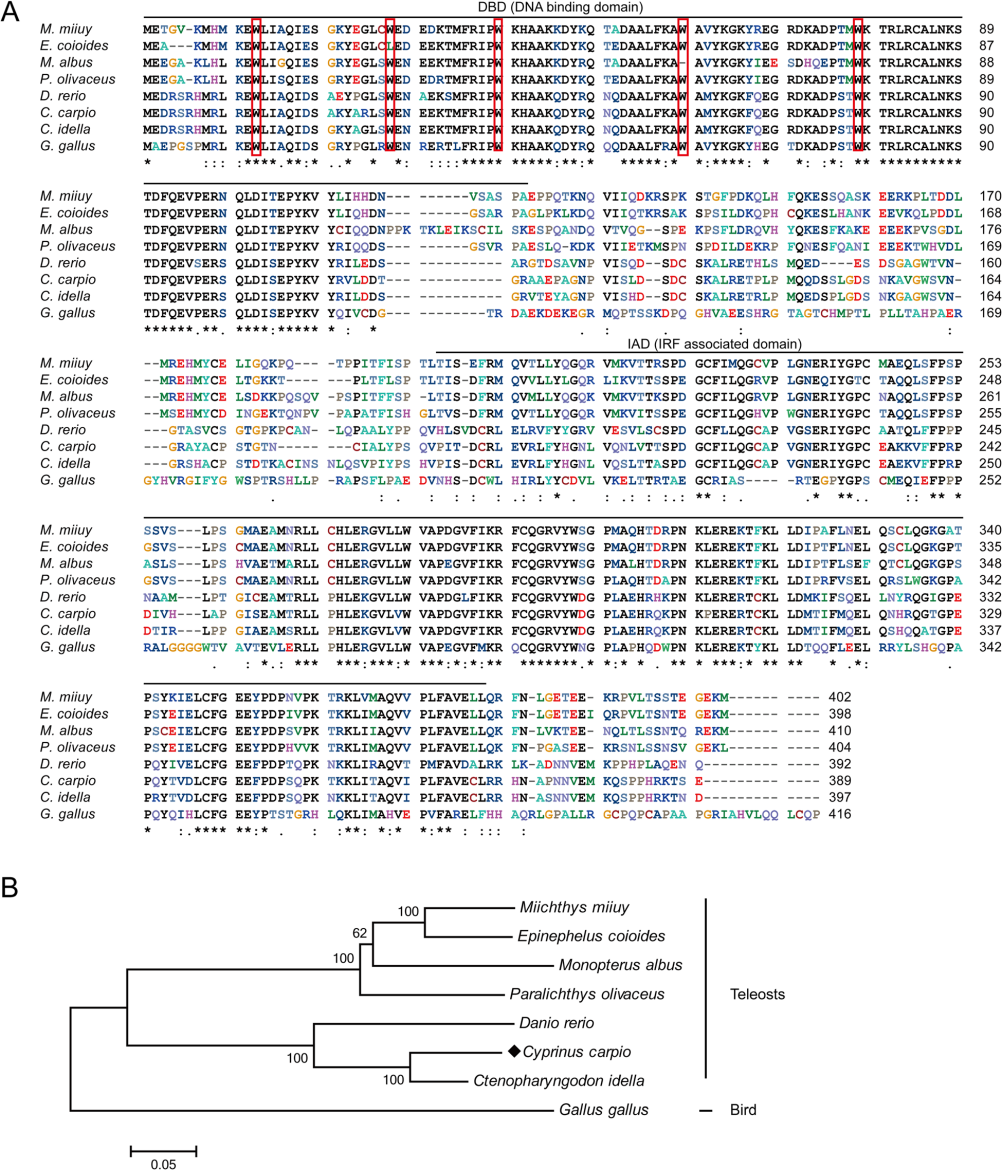
Multiple alignments (**a**) and phylogenetic analysis (**b**) of IRF10 protein sequences in different species. Identical (*) and similar (: or .) residues are indicated. The DBD and IAD are indicated by black lines. Five tryptophan (W) residues are boxed in red. The phylogenetic tree was produced by the neighbour-joining method in MEGA 5.0. *C. carpio* IRF10 is marked with a solid diamond (♦). The GenBank accession numbers of the genes are listed in Table 2

The predicted protein of *Cc*IRF10 shares 89.2% identity with that of *C. idella* IRF10, 52.7–75.6% identity with those of other teleost IRF10s and 50.1% identity with a bird (*G. gallus*) protein (Table 2). Multiple alignments of *Cc*IRF10 with the amino acid sequences from other vertebrates revealed areas of amino acids conserved in all vertebrates. Significant homology was found in the putative DBD (Fig. 1a). To explore the phylogenetic relationships of IRF10 in vertebrates, a phylogenetic tree including IRF10s from all known species was constructed using the neighbour-joining method. The tree was divided into two branches, teleost and bird. *Cc*IRF10 had the closest relationship with *C. idella* IRF10 (Fig. 1b).

**Table 2:** Protein length and GenBank accession numbers of IRF10

Species	Protein length	GenBank accession No.
*Cyprinus carpio*	389	MT646905
*Ctenopharyngodon idella*	397	ACT83676
*Danio rerio*	392	ABY91290
*Epinephelus coioides*	398	AKC01040
*Monopterus albus*	410	AKB09095
*Miichthys miiuy*	402	AHB59741
*Paralichthys olivaceus*	404	BAI63219
*Gallus gallus*	416	AAK55444

### Tissue distribution of *Cc*IRF10

**Fig. 2:**
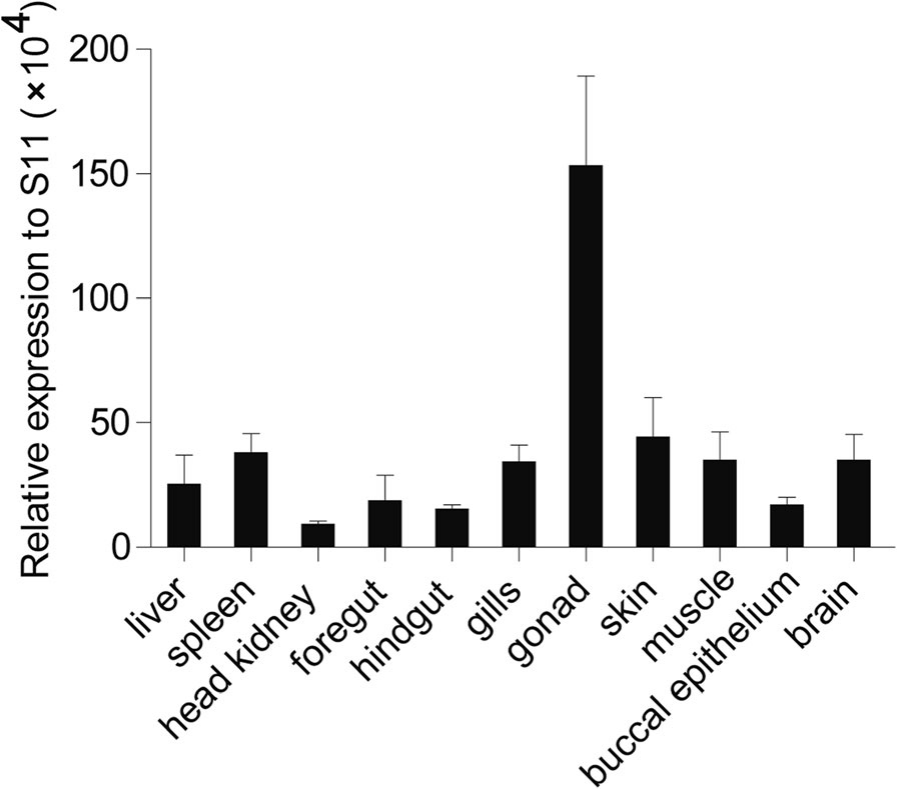
Tissue-specific expression of *Cc*IRF10 under normal physiological conditions. *Cc*IRF10 mRNA expression in the liver, spleen, head kidney, gills, skin, foregut, hindgut, buccal epithelium, gonad, muscle and brain was determined by real-time PCR. The gene expression levels were normalized using 40S ribosomal protein S11 mRNA. (*n* = 3)

The expression patterns of *Cc*IRF10 in 11 tissues of healthy common carp, including the liver, spleen, head kidney, foregut, hindgut, gills, gonad, skin, muscle, buccal epithelium and brain, were detected by real-time PCR. The results showed that *Cc*IRF10 mRNA was detected in all examined tissues, with the highest expression in the gonad, the lowest expression in the head kidney, and moderate expression in the other nine tissues (Fig. 2).

### Gene expression of *Cc*IRF10 in response to poly I:C and *A. hydrophila* stimulation in vivo

**Fig. 3:**
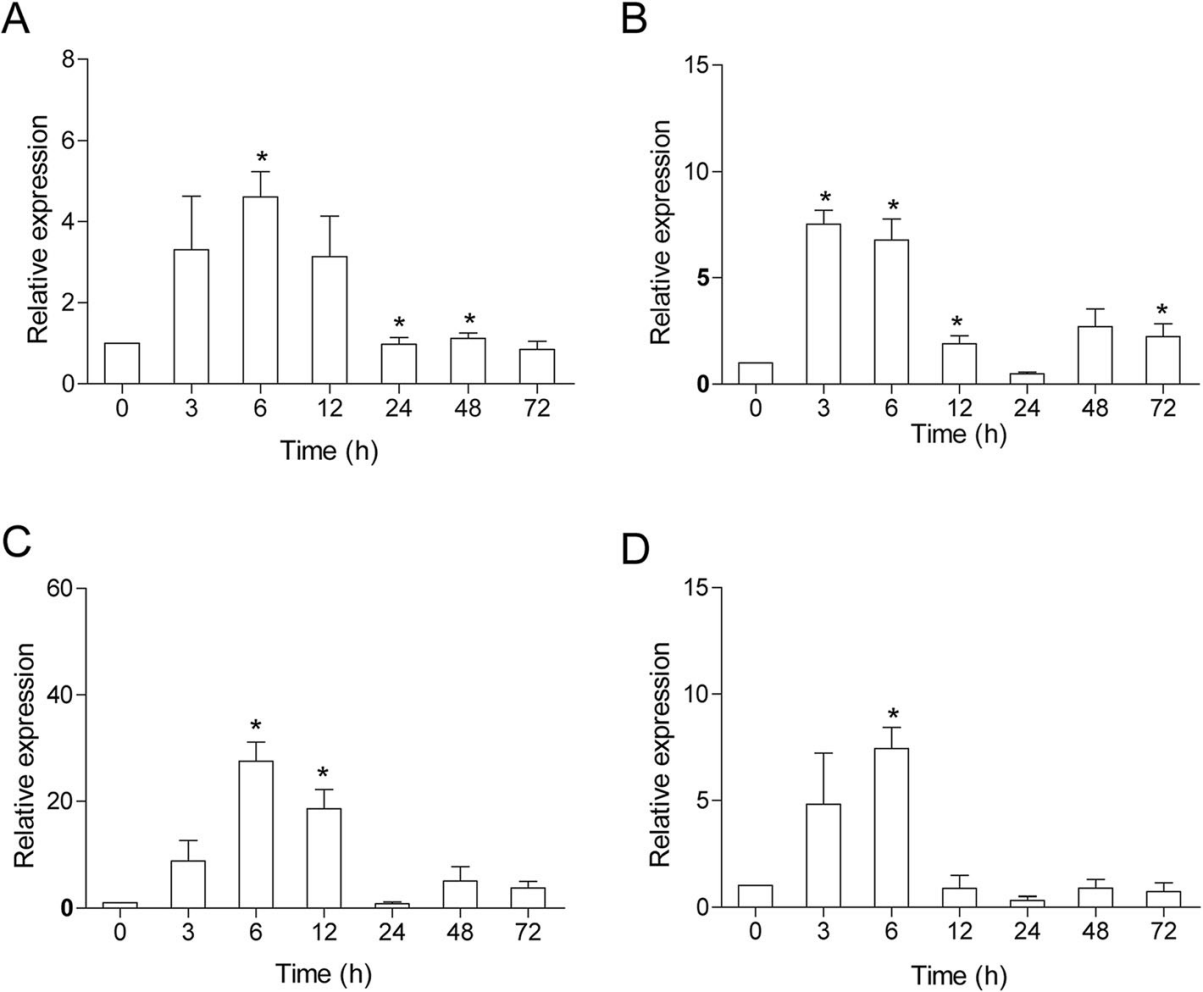
Expression analysis of *Cc*IRF10 in response to poly I:C challenge in vivo. Total RNA was extracted from spleen (**a**), head kidney (**b**), foregut (**c**) and hindgut (**d**) tissues at 0 (as a control), 3, 6, 12, 24, 48 and 72 h post injection for real-time PCR. The expression was normalized using that of the 40S ribosomal protein S11. (n = 3, mean ± SD, *P < 0.05)

To determine the function of *Cc*IRF10 in immune defence in common carp, *Cc*IRF10 expression was examined in some immune-related tissues after viral or bacterial challenge. Upon poly I:C stimulation, the peak expression of *Cc*IRF10 appeared at 6 hpi in the spleen (4.5-fold, *P* < 0.05), foregut (27.5-fold, *P* < 0.05) and hindgut (7.5-fold, *P* < 0.05). In the head kidney, *Cc*IRF10 expression peaked at 3 hpi (7.5-fold, *P* < 0.05) (Fig. 3).

**Fig. 4:**
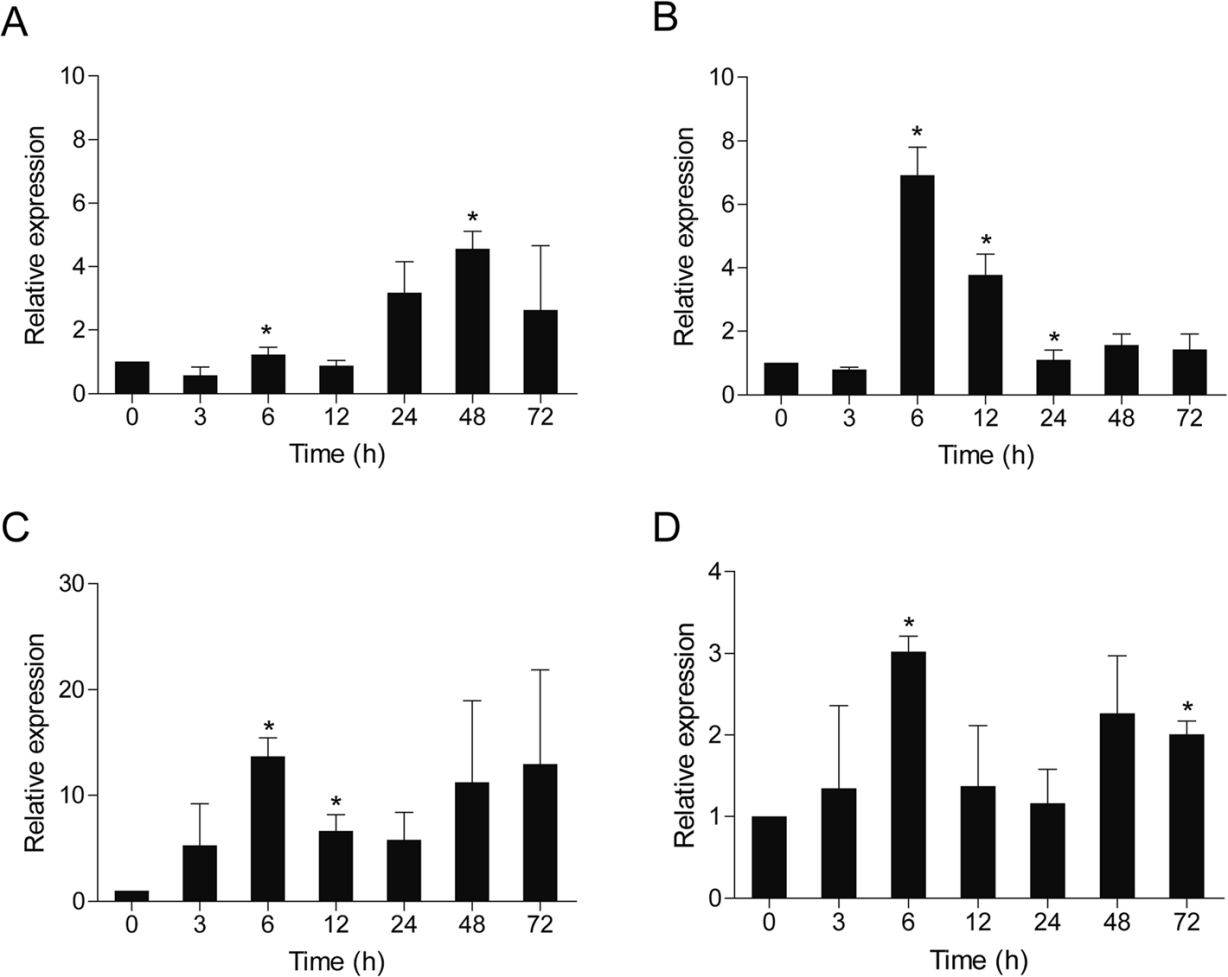
Expression analysis of *Cc*IRF10 in response to *A. hydrophila* challenge in vivo. Total RNA was extracted from spleen (**a**), head kidney (**b**), foregut (**c**) and hindgut (**d**) tissues at 0 (as a control), 3, 6, 12, 24, 48 and 72 h post injection for real-time PCR. The expression was normalized using that of the 40S ribosomal protein S11. (n = 3, mean ± SD, *P < 0.05)

In *A. hydrophila*-infected fish, *Cc*IRF10 was upregulated in the head kidney (6.8-fold, *P* < 0.05), foregut (13.7-fold, *P* < 0.05) and hindgut (3.0-fold, *P* < 0.05) at 6 hpi. In the spleen, *Cc*IRF10 reached its peak at 48 hpi, with a 4.6-fold induction (*P* < 0.05) (Fig. 4).

### Expression of *Cc*IRF10 upon poly I:C, LPS and PGN stimulation in vitro

**Fig. 5:**
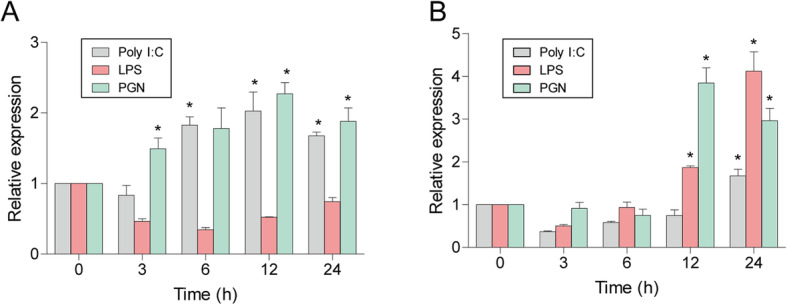
Expression levels of *Cc*IRF10 in PBLs (**a**) and HKLs (**b**) upon poly I:C and LPS stimulation. Cells were collected at 0 (as a control), 3, 6, 12 and 24 h post-infection for RNA extraction and real-time PCR analysis. The expression was normalized using that of the 40S ribosomal protein S11. (n = 3, mean ± SD, *P < 0.05)

To examine the *Cc*IRF10 transcription level in response to poly I:C, LPS or PGN challenge in vitro, we isolated leukocytes from the peripheral blood and head kidneys of *C. carpio*. As shown in Fig. 5a, real-time PCR data showed that the expression of *Cc*IRF10 in the PBLs was upregulated by poly I:C (2.0-fold, *P* < 0.05) and PGN (2.2-fold, *P* < 0.05) at 12 h, but not by LPS. In HKLs, *Cc*IRF10 was induced by all PAMPs. Upon PGN stimulation, the expression of *Cc*IRF10 reached its peak at 12 h (3.8-fold, *P* < 0.05), and after stimulation by poly I:C (1.7-fold, *P* < 0.05) and LPS (4.1-fold, *P* < 0.05), the highest expression was found at 24 h (Fig. 5b).

### mRNA expression of cytokines in EPC cells overexpressing *Cc*IRF10

**Fig. 6:**
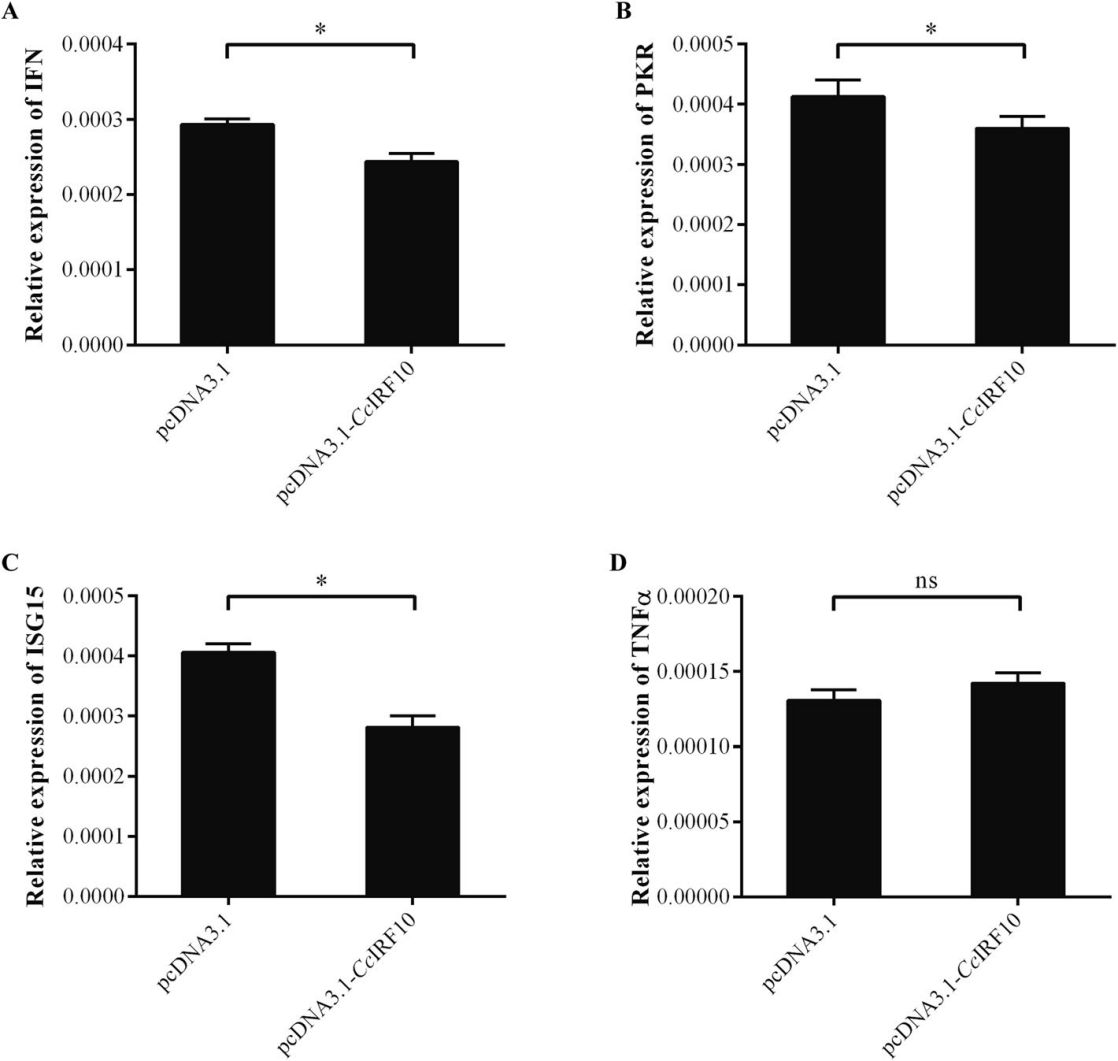
Relative expression of IFN (**a**), PKR (**b**), ISG15 (**c**) and TNFα (**d**) in *Cc*IRF10-transfected EPC cells. The expression was normalized to that of EF1α. (n = 3, mean ± SD, *P < 0.05)

To investigate the regulatory role of *Cc*IRF10 in the IFN signalling pathway, the gene expression of IFN, two IFN-stimulated genes (PKR and ISG15) and TNFα was detected after overexpression of *Cc*IRF10 in EPC cells (Fig. S2 and S3). The results showed that the gene expression of IFN, PKR and ISG15 was reduced in the cells transfected with pcDNA3.1-EGFP-*Cc*IRF10, which were 83, 0.87 and 0.69% of the expression in control cells, respectively (*P* < 0.05, Fig. 6a-c). However, the expression of TNFα, a non-ISG, was not changed in the cells (Fig. 6d).

## Discussion

IRF10, which has been reported to play important roles in immune responses to both viral and bacterial infections in teleosts, can inhibit the activation of IFN promoters and negatively regulate fish antiviral gene expression to prevent an excessive immune response [10, 11, 32]. In the present study, a non-mammalian IRF10 gene was cloned from common carp, and the antiviral and antibacterial immune functions of *Cc*IRF10 were investigated. The predicted *Cc*IRF10 protein contains two conserved functional domains, the N-terminal DBD and the C-terminal IAD, suggesting that the functions of IRF10 may be conserved throughout vertebrates. The DBD contains a highly conserved five-tryptophan repeat that can bind to IFN-stimulating response elements (ISREs) and IRF-regulatory elements (IRF-Es) in target promoters [4, 33]. Notably, the five well-conserved tryptophan residues in *D. rerio*, *C. idella*, *G. gallus*, and *C. carpio* are all at positions 10, 25, 37, 57 and 76 in the N-terminus. The IAD, which is responsible for homo−/heterodimer interactions of the IRFs and association with other transcription factors, is less conserved than the DBD [5]. Phylogenetic analysis of predicted IRF10 protein sequences of *C. carpio* and other vertebrate species supported the division of IRF10 into two branches: teleost and bird. These results match the established evolutionary relationships among teleost and other vertebrate species and support the authenticity of the nomenclature for IRF10.

IRF10 was first identified in *G. gallus* and is highly expressed in white blood cells and splenic lymphocytes, whereas low expression levels are found in other tissues [8]. In the present study, constitutive expression of the *Cc*IRF10 gene was detectable in all 11 tissues of *C. carpio* when analysed by real-time PCR, although there were differences in the levels of expression. This ubiquitous tissue expression pattern supports the findings of previous studies on IRFs in teleosts, including *M. albus* [17], *G. morhua* [13], *D. rerio* [34], *O. mykiss* [35], turbot (*Scophthalmus maximus*) [36], *P. olivaceus* [12, 37], rock bream (*Oplegnathus fasciatus*) [38], blunt snout bream (*Megalobrama amblycephala*) [39] and half-smooth tongue sole (*Cynoglossus semilaevis*) [40]. *Cc*IRF10 was found to be most highly expressed in gonads (Fig. 2), which is different from the expression patterns in *P. olivaceus*, *C. idella* and *M. albus*. In *P. olivaceus*, the expression of IRF10 has been found to be very high in the gills, intestine, trunk kidney, heart, stomach, head kidney and PBLs; in *C. idella*, IRF10 expression has been found to be high in all tested tissues, with the highest expression in the thymus and gills; and in *M. albus*, the highest expression level has been observed in intestine, whereas the lowest level has been found in the liver [12, 15, 17]. However, *D. rerio* IRF10 is highly expressed in the testis, which is also a reproductive organ [9]. These results suggest that IRF10 may not only play an important role in the immune system but also likely participate in the regulation of the reproductive system in teleosts.

Previous studies on *C. carpio* have shown that the expression of IRF1, IRF3, IRF5 and IRF7 is upregulated upon stimulation with poly I:C or viruses [25–27]. In the present study, following poly I:C injection, the induction of *Cc*IRF10 in the foregut (27.5-fold) was much stronger than that in the other tissues (4.5- to 7.5-fold), revealing the important role of *Cc*IRF10 in the mucosal immune system response to poly I:C (Fig. 3). Moreover, similar results were observed in PBLs and HKLs with poly I:C stimulation (Fig. 5). The expression of IRF10 in *P. olivaceus* is also upregulated by poly I:C stimulation in PBLs [12]. In *G. morhua*, two isoforms of IRF10 have been identified, and the expression of the long isoform reaches its peak at 6 hpi at 16 °C and 24 hpi at 10 °C, whereas the highest expression of the short isoform is observed at 6 hpi at both 16 °C and 10 °C after poly I:C stimulation [13, 41]. Moreover, IRF10 in *P. olivaceus*, *C. idella*, *M. albus* and zebrafish embryo fibroblast-like ZF4 cells can be upregulated by viruses (viral haemorrhagic septicaemia virus [VHSV] or grass carp haemorrhagic virus [GCHV]) or poly I:C [9, 12]. The observed induction of IRF10 expression by various viruses and poly I:C suggests that the fish IRF10 may play a crucial role in protecting the host from viral infection.

*A. hydrophila*, a well-known fish-pathogenic bacterium, is primarily found in temperate and freshwater environments and causes infections in various organisms. Fish are becoming increasingly susceptible to *A. hydrophila* because of the increasingly intensive rearing methods used in aquaculture [42]. Moreover, *A. hydrophila* breakouts have caused great economic losses around the world [43]. To gain insights into the immune mechanism of *Cc*IRF10 in the antibacterial response, its expression pattern in response to *A. hydrophila* was investigated using real-time PCR. When fish were challenged with *A. hydrophila*, the levels of *Cc*IRF10 were upregulated in all four tissues, with the highest induction in the foregut (Fig. 4). IRF10 of *P. olivaceus* can also be induced by *Edwardsiella tarda* and *Streptococcus iniae* [37]. Upon *Aeromonas salmonicida* infection, the greatest expression of the long isoform of *G. morhua* IRF10 occurs at 24 hpi, whereas the highest expression of the short isoform is observed at 6 hpi at 10 °C and 16 °C, suggesting the distinct roles of the two isoforms in the immune system of *G. morhua* [13, 41]. It should be noted that *E. coioides* IRF10 is responsive to both poly I:C stimulation and *Vibrio parahaemolyticus* infection but increases to a greater extent after poly I:C stimulation [10]. In accordance with these results, our study showed that the fold change in *Cc*IRF10 induced by poly I:C stimulation (4.5- to 27.5-fold) was greater than that induced by *A. hydrophila* infection (3.0- to 13.7-fold). LPS is the major component of the outer membranes of gram-negative bacteria, but it has been reported that lower vertebrates (such as fish) may be resistant to the toxic effects of LPS [44]. Therefore, it was unsurprising that *Cc*IRF10 was not upregulated by LPS in the PBLs (Fig. 5a). PGN, a major component of the bacterial cell wall, consists of sugars and amino acids. Similar to the findings of a previous study regarding head kidney macrophages of *O. mykiss*, *Cc*IRF10 was upregulated by PGN stimulation in vitro [15]. Hence, our in vivo and in vitro findings, together with the previous analogous results, suggest that *Cc*IRF10 is more susceptible to poly I:C than to *A. hydrophila* infection and plays a substantial role in the foregut, which is a mucosal immune organ. Moreover, fish IRF10 may play essential roles in both antiviral and antibacterial defence, as reported for *G. gallus* IRF10.

In mammals, IFNs are natural glycoproteins produced by cells of the innate and adaptive immune systems in most vertebrates in response to challenge by viruses, bacteria, fungi, parasites, and tumour cells [45]. In addition, IFNs can also be produced by non-immune cells such as fibroblasts and epithelial cells [45]. Similarly, in fish, IFNs play a crucial role in innate immunity [46, 47]. To investigate the regulatory role of *Cc*IRF10 in the IFN response, we detected the mRNA expression of type I IFN, ISGs (PKR and ISG15) and TNFα in *Cc*IRF10-transfected EPC cells. The transcription of IFN, PKR, ISG15 was downregulated in EPC cells transfected with the pcDNA3.1-*Cc*IRF10 plasmid (Fig. 6). This result is in line with the findings of a previous study that overexpression of *D. rerio* IRF10 downregulated the expression of IFN-stimulated genes induced by poly I:C and promoted the replication of spring viremia of carp virus (SVCV) in EPC cells [9]. Moreover, this study found that the ISRE site in the promoter was responsible for *Dr*IRF10-mediated inhibition of gene expression of IFNs and ISGs [9, 48]. The mechanism involved in the inhibition of IFN signalling pathway in common carp may be similar, which needs our further study. However, *G. gallus* IRF10 can upregulate the expression of MHC class I and GBP [8], suggesting that the function of IRF10 might be different between fish and birds.

## Conclusions

In the present study, the full-length cDNA sequence of IRF10 from common carp was identified and characterized. In vivo and in vitro studies indicated that *Cc*IRF10 participates in both antiviral and antibacterial immune responses. Furthermore, overexpression of *Cc*IRF10 was able to decrease the expression of the IFN and IFN-stimulated genes PKR and ISG15, indicating that *Cc*IRF10 might negatively regulate the IFN response of *C. carpio*. The study will provide a valuable experimental foundation for future studies on the immune system of common carp and a theoretical basis for the prevention of fish disease.

## Supplementary Information

Additional file 1: **Supplementary Fig. S1.** Nucleotide sequence and the deduced amino acid sequence of *C. carpio* IRF10. Lowercase letters indicate the 5′ and 3′ UTR, while uppercase letters indicate the ORF or amino acid. The start codon (ATG) and stop codon (TGA) are boxed in red. The DBD and IAD are shaded in blue and pink, respectively. The NLS is underlined, and the five tryptophan (W) residues are boxed in black.

Additional file 2: **Supplementary Fig. S2.** Overexpression of *Cc*IRF10 in EPC cells. EPC cells were transfected with pcDNA3.1-EGFP empty plasmid or pcDNA3.1-EGFP-*Cc*IRF10, and the gene expression of *Cc*IRF10 was detected using real-time PCR. The expression was normalized to that of EF1α. (*n* = 3, mean ± SD, **P < 0.01).

Additional file 3: **Supplementary Fig. S3.** Transfection efficiency of EPC cells. Bright field (A) and fluorescent image (B) of EPC cells transfected with pcDNA3.1-EGFP empty plasmid. Bright field (C) and fluorescent image (D) of EPC cells transfected with pcDNA3.1-EGFP-*Cc*IRF10 plasmid. (original magnification × 40).

## Data Availability

The dataset supporting the conclusions of this article is available in the GenBank (https://www.ncbi.nlm.nih.gov/nuccore/MT646905) and the accession number is MT646905.

## Abbreviations

IFN: Interferon
IRF: Interferon regulatory factor
ISG: IFN-stimulated gene
DBD: DNA-binding domain
NLS: Nuclear localization signal
IAD: IRF associated domain
poly I:C: polyinosinic:polycytidylic acid
LPS: Lipopolysaccharide
EPC: Epithelioma papulosum cyprinid
RACE: Rapid amplification of the cDNA ends
SMART: Simple modular architecture research tool
PBL: Peripheral blood leucocyte
HKL: Head kidney leukocyte
PGN: Peptidoglycan
ORF: Open reading frame
ANOVA: One-way analysis of variance
UTR: Untranslated region
hpi: Hour post injection
ISRE: IFN stimulating response element
IRF-E: IRF regulatory element
VHSV: Viral hemorrhagic septicemia virus
GCHV: Grass carp haemorrhagic virus
SVCV: Spring viremia of carp virus
RLR: Retinoic acid-inducible gene I (RIG-I)-like receptor
