# Supplementary figures and images for: Identification of an IRF10 gene in common carp (Cyprinus carpio L.) and analysis of its function in the antiviral and antibacterial immune response
Source: BMC Vet Res. 2020 Nov 19;16:450. doi: 10.1186/s12917-020-02674-z (PMC7678311; doi:10.1186/s12917-020-02674-z)

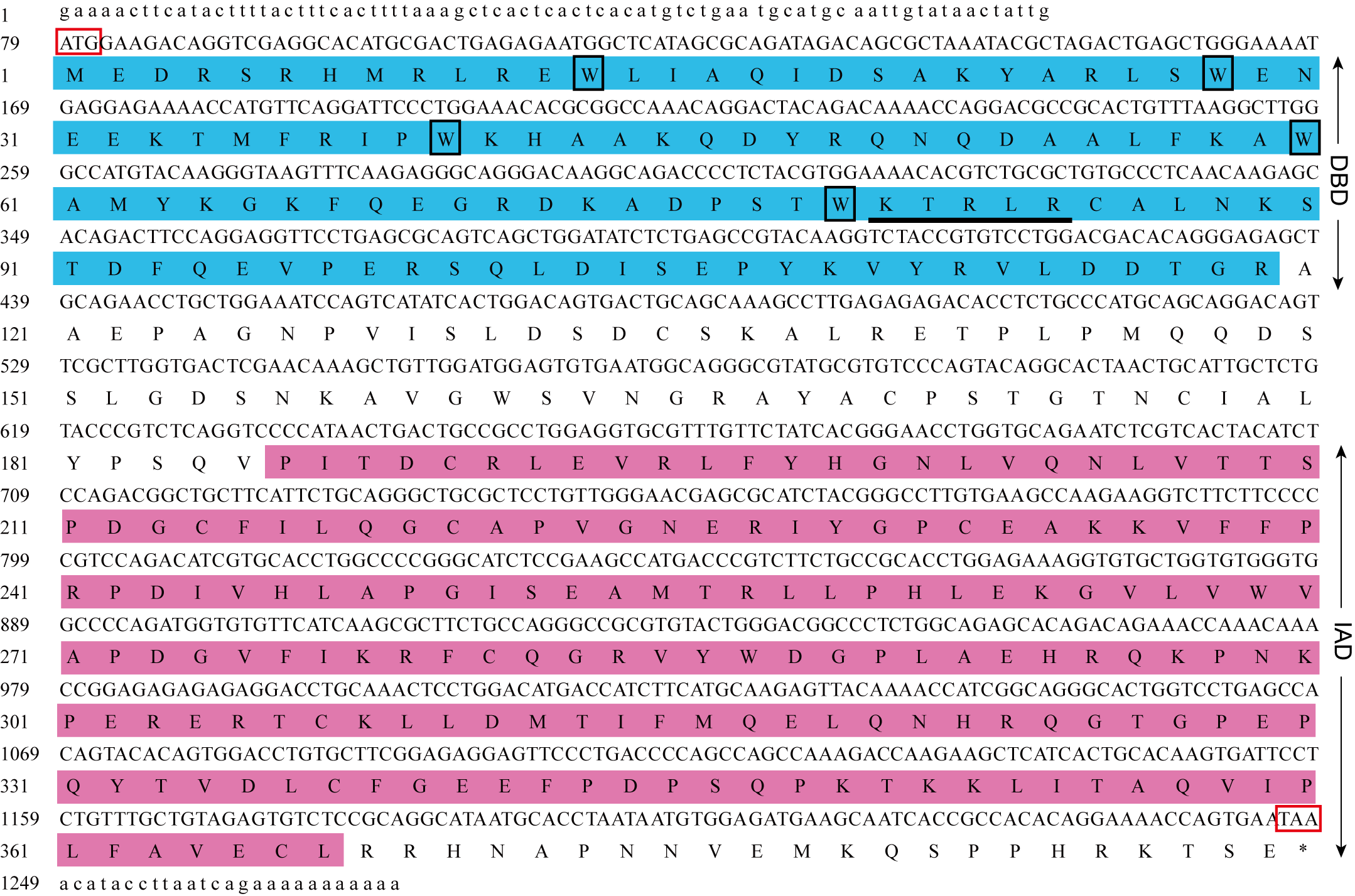

Supplement: Supplementary file 1 — Supplementary Fig. S1. Nucleotide sequence and the deduced amino acid sequence of C. carpio IRF10. Lowercase letters indicate the 5′ and 3′ UTR, while uppercase letters indicate the ORF or amino acid. The start codon (ATG) and stop codon (TGA) are boxed in red. The DBD and IAD are shaded in blue and pink, respectively. The NLS is underlined, and the five tryptophan (W) residues are boxed in black. [file 12917_2020_2674_MOESM1_ESM.tif]

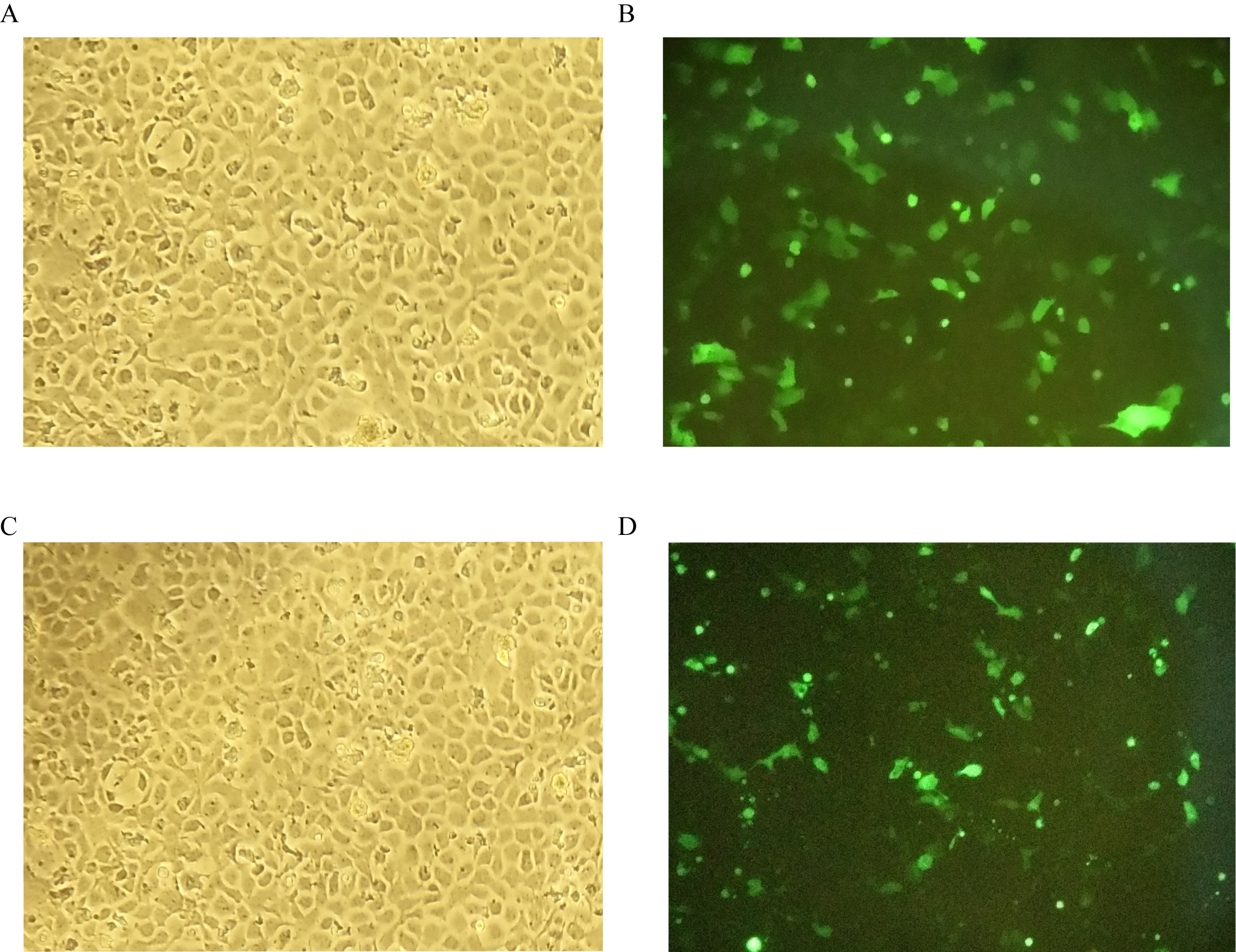

Supplement: Supplementary file 3 — Supplementary Fig. S3. Transfection efficiency of EPC cells. Bright field (A) and fluorescent image (B) of EPC cells transfected with pcDNA3.1-EGFP empty plasmid. Bright field (C) and fluorescent image (D) of EPC cells transfected with pcDNA3.1-EGFP-CcIRF10 plasmid. (original magnification × 40). [file 12917_2020_2674_MOESM3_ESM.tif]
